# Network Analysis of miRNA and mRNA Changes in the Prelimbic Cortex of Rats With Chronic Neuropathic Pain: Pointing to Inflammation

**DOI:** 10.3389/fgene.2020.00612

**Published:** 2020-06-23

**Authors:** Guohong Cai, Yuanyuan Zhu, Yan Zhao, Jing Chen, Chihua Guo, Feifei Wu, Jing Huang, Shengxi Wu

**Affiliations:** ^1^Department of Neurobiology, School of Basic Medicine, Fourth Military Medical University, Xi’an, China; ^2^Department of Gastroenterology, First Affiliated Hospital of Xi’an Jiaotong University, Xi’an, China; ^3^Department of Anatomy, School of Basic Medicine, Fourth Military Medical University, Xi’an, China; ^4^Institute of Basic Medical Sciences, Xi’an Medical University, Xi’an, China; ^5^Basic Medicine Teaching Experiment Center, School of Basic Medicine, Fourth Military Medical University, Xi’an, China

**Keywords:** neuropathic pain, prelimbic cortex, microRNA, mRNA, microglia, inflammation

## Abstract

Neuropathic pain (NP) is a complex, chronic pain condition caused by injury or dysfunction affecting the somatosensory nervous system. This study aimed to identify crucial mRNAs and microRNAs (miRNAs) in the prelimbic cortex (PL) of NP rats. mRNA and miRNA microarrays were applied in the present study. The miRNA-mRNA regulatory network was constructed by using ingenuity pathway analysis (IPA). A total of 35 differentially expressed (DE) RNAs (24 miRNAs and 10 mRNAs) were identified in the spared nerve injury (SNI) group compared with the control group. The DE miRNA-mRNA network showed that IL-6 and tumor necrosis factor (TNF) were core components. Mir-30c-5p and mir-16-5p were the most connected miRNAs in the network. Interestingly, four mRNAs (Rnase 4, Egr2, Rexo4, and Klf2) with significantly increased expression were abundantly expressed in microglia, which was verified by the real-time quantitative polymerase chain reaction (qPCR). Furthermore, the expression of Rnase4 and Egr2 decreased in M1-polarized macrophages and increased in M2-polarized macrophages. In conclusion, we screened dozens of DE mRNAs and miRNAs in the PL of SNI rats. The core of the DE mRNA and miRNA network pointed to molecules associated with inflammation. Four mRNAs (Rnase4, Egr2, Rexo4, and Klf2) might be the potential markers of M2 polarization.

## Introduction

One-fifth of the world’s population suffers from chronic pain (Reid et al., 2011), which is accompanied by many comorbidities, including mood disorders, deficits in cognition and memory, inattention and decreased motivation (Simons et al., 2014). Clinically, a large percentage of patients with chronic pain are accompanied with anxiety (Dersh et al., 2002). Similarly, patients with anxiety usually show more pain symptoms and cognitive impairment (Bair et al., 2003). Due to the interactions between pain and anxiety, the treatment of chronic pain becomes more challenging. Moreover, the specific molecular mechanism involved in chronic pain-induced anxiety is not yet fully understood. Unfortunately, as of now, the specific mechanism of chronic pain-induced anxiety remains unclear.

In chronic pain, pain signals are transmitted to several brain regions, which are thought to be involved in the initiation of anxiety (Alba-Delgado et al., 2013). We previously showed the role of the anterior cingulate cortex (ACC) in affective responses to pain and related anxiety-like behavior (Guo et al., 2017). Functional imaging studies suggested that in addition to the ACC, the prefrontal cortex (PFC) was also involved in pain-related mood disorders (Seifert and Maihofner, 2009). The medial PFC (mPFC) has been shown to play an important role in pain-related perception and emotional processes (Hung et al., 2014). In a chronic pain animal model, Metz et al. (2009) reported the abnormalities in the function and morphology of the mPFC. The prelimbic cortex (PL), a subregion of the mPFC, might be a critical region in the pathological process of pain-induced anxiety (Wang et al., 2015). The pyramidal cells in different layers of the PL from neuropathic pain mice exhibited different excitability (Mitric et al., 2019). However, the identification of more molecular mechanisms is needed.

MicroRNAs (miRNAs) are noncoding genes that contain 19–25 nucleotides, which play a pivotal role in controlling biological processes by affecting messenger RNA (mRNA) stability and protein translation (Lopez-Gonzalez et al., 2017). There are a large number of reports of miRNA changes in the spinal cord of neuropathic pain models (Gong et al., 2015; Liu et al., 2018; Cao et al., 2019), whereas only a few studies have examined the mPFC (Li et al., 2019). We hypothesized that changes in miRNA and mRNA expression in the PL might influence behavioral changes in neuropathic pain and will become potential targets for pain control therapy. Based on this evidence, in this study, we sought to determine the key changes in miRNA and mRNA expression in PL that might influence neuropathic pain and related anxiety.

## Materials and Methods

### Animals

Male Sprague-Dawley (SD) rats (200–250 g, 6–7 weeks of age) were acquired from the Experimental Animal Center of Medical University. All experimental animals were housed in groups in a temperature- and humidity-controlled room with a 12:12 h light-dark cycle and water available ad libitum. All experimental procedures were approved by the Institutional Animal Care and Use Committee (IACUC) at the University and conformed to the Guide for the Care and Use of Laboratory Animals published by the National Institutes of Health (NIH). Every effort has been made to minimize animal suffering and reduce the number of animals used.

### Surgery

Spared nerve injury surgery was performed as described (Decosterd and Woolf, 2000). Briefly, under pentobarbital anesthesia, the tibial branches and sural common peroneal of the left sciatic nerve were exposed. Then, the tibial and common peroneal nerves were transected, while the sural nerve was kept intact. For the sham group, nerves were exposed but not transected.

### Behavioral Testing

#### Mechanical Allodynia Test

Mechanical allodynia was measured by the paw withdrawal threshold (PWT) in response to a series of von Frey hairs ranging from 0.4 to 60 g. Rats were placed individually into Plexiglas chambers on an elevated wire-mesh floor, and the von Frey hairs were applied to the plantar surface of the hind paw with increasing forces until the rat withdrew. The lowest force that produced at least 3 withdrawal responses in five consecutive applications was defined as the PWT.

#### Open-Field Test

Rats were brought into the test room and allowed them to acclimate for at least half an hour before testing. The open-field (OF) arena was a black plastic box measuring 100 × 100 × 40 cm, and the test was performed under full-light conditions (1000 lux). Rats were placed in the center of the arena and their behavior was recorded for 5 min from above. After the test, rats were returned to their cages and the arena was wiped with ethanol. The automated analysis system (SMART 3.0, Panlab S.L.U.) was used to calculate the time spent in the center of the arena.

### Lesions of the Prelimbic Cortex

The specific steps were mainly referring to the previous reference (Wang et al., 2015). On day 7 after surgery, the rats were anesthetized with pentobarbital, 300 mg per 100 g intraperitoneally, and positioned in a stereotaxic instrument. The injection of prelimbic cortex (PL) was according to the atlas of Paxinos and Watson (1997) (AP, +2.8, ML, ±0.5, DV, −4 mm). A 0.5 μl volume of a 0.1-M solution of quinolinic acid (QA), in phosphate buffered saline (0.1 mol/L, pH = 7.4) was administered in each side of PL in 1 min (0.5 μl/min). The rats in control group were injected with saline in the same way. After the surgery, the animal was returned to its home cage. Seven days after the surgery of lesion, OF and PWL were assessed. After the behavior tests, the location and effect of the injection were detected by immunofluorescence. The labeling of glial fibrillary acidic protein (GFAP) or neuronal nuclei protein (NeuN) was performed on free floating 40 μm thick transverse sections from rats perfused with 4% paraformaldehyde 7 days following injection. The sections were incubated with rabbit anti-GFAP (1:300, Dako) or anti-NeuN (1:500, Abcam) for 15 h at 4°C. Secondary antibody was donkey anti-rabbit Alexa Fluor 488 (1:1000, Jackson). Sections were counterstained with 4′, 6-diamidino-2-phenylindole (DAPI). Images were captured with an Olympus FV1000 confocal scanning laser microscope with appropriate laser scanning and filter for Alexa 488.

### mRNA and miRNA Microarray Analysis

On day 14 after surgery, rats were anesthetized with pentobarbital, 300 mg per 100 g intraperitoneally, and were decapitated immediately. The brains were rapidly removed, and the contralateral PL regions of the brains were dissected. Three samples of each group were tested for mRNA and miRNA microarray analysis. PL tissue RNA was isolated with the TRIzol reagent (Thermo Fisher Scientific, Inc.), according to the manufacturer’s protocol. Affymetrix GeneChip Rat Genome 230 Array 2.0 and GeneChip^®^ Rat miRNA 4.0 Array (Affymetrix; Thermo Fisher Scientific, Inc.) were applied in the present study and all associated procedures were conducted by GeneChem Co., Ltd., (Shanghai, China), according to the standard operating procedure. All data were corrected by RMA method. Then, log2 transformation along with quantile normalization was applied subsequently. Expressional data were analyzed in R software using the Limma package to identify differentially expressed genes. The microarray data was deposited in the GEO repository (GSE145226, mRNA) and (GSE145199, miRNA).

### Network Analysis and Visualization

The interactome of differentially expressed (DE) mRNAs and miRNAs was generated using the Ingenuity Pathway Analysis software (Ingenuity Systems^1^). Only experimentally observed microRNA-target interactions and those predicted with high confidence were used. IPA assigns “high confidence” to interactions involving a conserved or highly conserved miRNA as defined by TargetScan (Lewis et al., 2005; Garcia et al., 2011; Osterndorff-Kahanek et al., 2018). In addition, we limited the network data to genes related to anxiety, anxiety disorders and discomfort.

### Cell Culture Treatments

The N9 cell line was purchased by ATCC (American Type Culture Collection). The N9 cells were plated on 6-well culture dishes at a density of ∼1 × 10^5^/cm^2^. The N9 cells were maintained in culture in DMEM, 10% heat-inactivated FBS, 1% penicillin & streptomycin Pen Strep (Gibco), and 1% glutamine (Sigma). First, the N9 cells were washed three times with PBS. Then, the N9 cells were incubated 24 h with LPS (25 ng/ml) (L4391, Sigma) for M1 polarization, and the cells were incubated 24 h with IL-4 (20 ng/ml) (SRP4137, Sigma) for M2 polarization.

### qRT-PCR Analysis

The N9 cells were lysed by TRIzol lysis buffer. Then, the total RNA from lysis buffer was isolated using a RNAprep Pure Cell kit (Tiangen) according to the manufacturer’s instructions. The total RNA from PL tissues was isolated using a tissue total RNA extraction kit (Tiangen, DP431) according to the manufacturer’s instructions. The specific experimental steps are as described in the previous article (Zhu et al., 2020). The mRNA was retrotranscribed into cDAN a total of 1 μg using a PrimeScriptTM RT reagent Kit (TaKaRa). Target cDNA levels were determined by RT-PCR (Thermo Fisher Scientific, Wilmington, MA, United States) using TB Green kit (TaKaRa) according to the manufacturer’s instructions. The amplification assays were performed in 25 μL reaction mixtures containing 2 × TB Green Premix. PCR was performed using 2 μL of the cDNA solution, 12.5 μL of TB Green mix, 1 μL of each primer (10 μM), and 8.5 μL of ddwater. The PCR profile was 1 min at 95°C, 45 cycles of 5 s at 95°C, and 20 s at 60°C. The cDNA was normalized with GAPDH. The forward and reverse PCR primers were as follows:

TNFα: 5′-ACACCATGAGCACAGAAAGC-3′ and 5′- GCC ACAAGCAGGA ATGAGAAG-3′;

IL-1β: 5′- TCTCGCAGCAGCACATCAAC-3′ and 5′- ACCA GCAGGTTATCAT CATCATCC;

iNOS: 5′- CCCTTCAATGGTTGGTACATGG-3′ and 5′- ACA TTGATCTCCGTG ACAGCC-3′;

IL-10: 5′-CTCTTACTGACTGGCATGAGGATC-3’ and 5’-AAGGAGTCGGTT AGCAGTATGTTG-3′;

Arg-1: 5′-CTCCAAGCCAAAGTCCTTAGAG-3′ and 5′- AG GAGCTGTCAT TAGGGACATC-3′;

Rnase4: 5′-CCAGTGCAAACGCTTCAACA-3′ and 5′-TGA CAACTCGCCT AGTGCTG-3′;

Egr2: 5′-CTCAGTGGTTTTATGCACCAGC-3′ and 5′-GAT GGGAGCGAAGC TACTCG-3′;

Rexo4: 5′-GCCTATCCGGAAGCTCGTTA-3′ and 5′-CTTG CAGCGCCT TCCAATTT-3′;

Klf2: 5′-CTCAGCGAGCCTATCTTGCC-3′ and 5′-CCAGTC CCATG GACAGGATG-3′.

### Western Blot Analysis

The experimental process is as described in the previous study (Guo et al., 2017). PL tissues of SNI rats and control rats were collected and lysed in 100–300 ml of radio immunoprecipitation assay (RIPA) lysis buffer. The BCA protein assay (Pierce) was used to quantify the total protein samples (20–40 mg), and then the samples were resolved via sodium dodecyl sulfate polyacrylamide gel electrophoresis (SDSPAGE) and transferred to PVDF membranes. The primary antibodies were as follows: mouse anti-β-actin (1:1,000, Cell Signaling Technology); rabbit anti-Rnase4 (1:1,000, Abcam); rabbit anti-Egr2 (1:1,000, Millipore). The immunoblots were incubated with the following secondary antibodies: horseradish peroxidase (HRP)-conjugated donkey anti-mouse IgG, HRP-conjugated donkey anti-rabbit IgG (1:5,000, Invitrogen). The enhanced chemiluminescence (ECL) detection method (Advansta) was used to visualize all Western blots. Image J software (version 1.47) was used to quantify the scanned images.

### Statistical Analyses

The data are expressed as the means ± SEM. Testing of statistical significance was performed using the unpaired *t*-test, two-sided *t*-test. Statistical significance was set to *p* < 0.05. *P* values were classified as ^∗^*p* < 0.05, ^∗∗^*p* < 0.01, ^∗∗∗^*p* < 0.001.

## Results

### The SNI Model Induces Mechanical Allodynia and Anxiety-Like Behaviors

**FIGURE 1:**
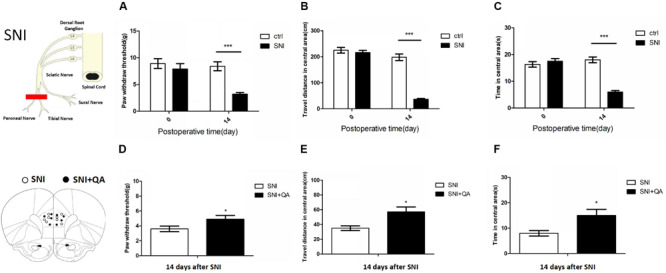
The SNI model of neuropathic pain induces mechanical allodynia and enhances anxiety-like behaviors. **(A)** Fifty percent of mechanical thresholds of mechanical allodynia were determined using von Frey filaments before surgery (baseline) and 14 days after surgery. (*n* = 8 rats per group; ***P < 0.001, two-tailed *t*-test). **(B,C)** Anxiety-like behaviors assessed in an OF test (*n* = 8 rats per group; two-tailed *t*-test). ***P < 0.001. **(D–F)** Paw withdrawal threshold and open field of the SNI rats after bilateral lesion of the PL by quinolinic acid, respectively (*n* = 8 rats per group; *P < 0.05, two-tailed *t*-test). The data are presented as mean ± s.e.m.

Rats in SNI group showed decreasing mechanical thresholds in the von Frey test for 14 days, which indicated robust mechanical allodynia (Figure 1A). In addition, anxiety-related exploratory behavior of rats was assessed by OF test 14 days after surgery. Compared with the control group, SNI rats traveled shorter distances and spent significantly less time in the center area of OF test (Figures 1B,C). These data showed that SNI induced long-term persistent nociceptive sensitization and increased in anxiety-like behaviors.

According to a previous study, the PL was the specific subregion of mPFC that was responsible for pain and related anxiety (Wang et al., 2015). We bilaterally infused quinolinic acid (QA), a potent neurotoxic compound, into the PL to damage it (Supplementary Figure S1). After bilateral lesion of the PL, SNI rats exhibited increased paw withdraw threshold and increased travel distance and time in the center area of the OF (Figures 1D–F), suggesting the vital role of the PL in modulating pain sensation and related anxiety-like behaviors.

### Differentially Expressed (DE) mRNAs and miRNAs

**FIGURE 2:**
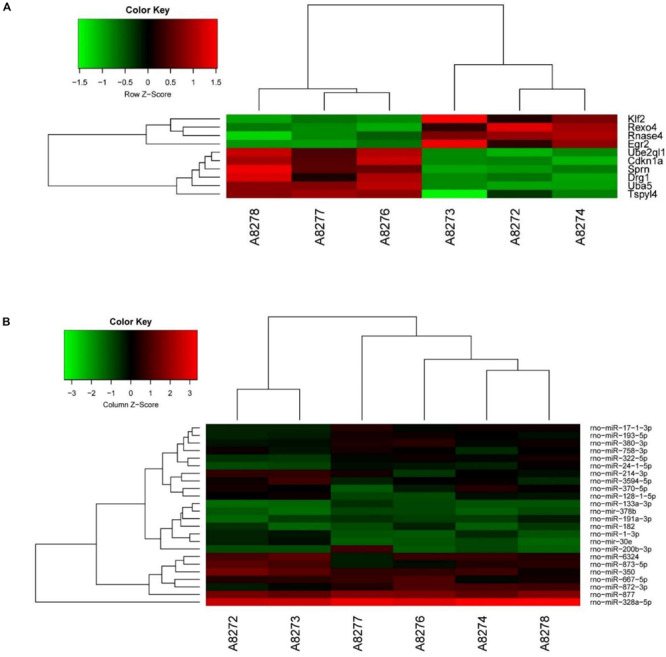
The clustering map and network analysis of DE mRNAs and miRNAs. **(A)** There were 4 up-regulated mRNAs and 6 down-regulated mRNAs. **(B)** There were 25 DE miRNAs (10 up-regulation and 15 down-regulation).

We analyzed DE mRNAs in the PL using a significance analysis of the GeneChip^®^ Rat Genome 230 2.0 Array, following the criteria log2-fold-change >1.5 and *p* < 0.05. Figure 2A shows the clustering map of the DE mRNAs. The information on the upregulated and downregulated mRNAs in the SNI group compared with the control group on 14 days after SNI has been shown in Table 1. There were four mRNAs with upregulated expression and six mRNAs with downregulated expression in the SNI group compared to the control group.

**TABLE 1:** The top 10 upregulated and downregulated differentially expressed genes (DEGs).

Gene symbol	Gene_location	Fold change	*P*-value	Regulation
Rexo4	chr3:5849502–5850253	2.0487504	0.006235537	up
Klf2	chr16:17941333–17942639	1.8269434	0.013266017	up
Rnase4	chr15:27089667–27090445	2.1179023	0.001291831	up
Egr2	chr20:21883885–21888177	3.1384263	0.007462819	up
Tspyl4	chr20:38640484–38641266	–1.913118	0.006247621	down
Ube2ql1	chr17:3739696–3740380	–1.534941	0.001901304	down
Sprn	chr1:199953670–199953768	–1.5222503	0.004164148	down
Uba5	chr8:109108739–109115887	–1.9610097	9.06281*E*−05	down
Drg1	chr14:83857802–83860049	–1.6962591	0.008923944	down
Cdkn1a	chr20:7379385–7385634	–1.5424892	0.00128275	down

We analyzed the DE miRNAs in the PL using significance analysis of the GeneChip^®^ Rat miRNA 4.0 Array using the criterion of log2-fold-change >1.5. Figure 2B shows the clustering map of DE miRNAs. There were 24 DE miRNAs (9 upregulated and 15 downregulated) in the rat PL on 14 days in the SNI group compared to the control group. Information on the miRNAs with upregulated and downregulated expression in the SNI group has been shown in Table 2.

**TABLE 2:** The detail information of the up-regulated and down-regulated miRNAs.

miRNA	Alignments	Fold change	Regulation
rno-miR-873-5p	5:53454961–53454981 (−)	1.842379163	up
rno-miR-370-5p	6:134210206–134210229 (+)	1.670982935	up
rno-mir-30e	5:141365115–141365206 (−)	1.668093704	up
rno-miR-3594-5p	10:109476519–109476539 (−)	1.610032711	up
rno-miR-128-1-5p	13:40907589–40907609 (+)	1.600926048	up
rno-miR-1-3p	18:2191683–2191704 (−)	1.595218818	up
rno-miR-6324	11:36339331–36339353 (−)	1.546903643	up
rno-miR-214-3p	13:77916255–77916275 (+)	1.514865807	up
rno-miR-350	13:92545090–92545113 (−)	1.513648268	up
rno-miR-24-1-5p	17:7351012–7351033 (−)	2.109342448	down
rno-miR-322-5p	X:140000212–140000233 (−)	1.979034368	down
rno-miR-872-3p	5:114979254–114979275 (+)	1.952591289	down
rno-miR-380-3p	6:134391034–134391054 (+)	1.87513786	down
rno-miR-200b-3p	5:172899014–172899036 (−)	1.744825387	down
rno-miR-17-1-3p	15:99853785–99853806 (+)	1.663682346	down
rno-miR-191a-3p	8:113614416–113614437 (+)	1.662845351	down
rno-miR-667-5p	6:134400514–134400537 (+)	1.640664214	down
rno-miR-758-3p	6:134392042–134392063 (+)	1.614745351	down
rno-miR-133a-3p	18:2189230–2189251 (−)	1.612216322	down
rno-miR-193-5p	10:65901067–65901088 (+)	1.590683342	down
rno-miR-877	20:2958184–2958203 (+)	1.563840454	down
rno-mir-378b	5:5156348–5156431 (−)	1.560021234	down
rno-miR-182	4:57071298–57071322 (−)	1.534569869	down
rno-miR-328a-5p	19:35122612–35122630 (−)	1.527511597	down

### Functional Prediction of DE miRNAs in SNI

To determine the functional roles of the miRNAs and their target genes in biological pathways, we performed gene ontology (GO) (Young et al., 2010) and KEGG pathway enrichment analysis for biological functions.

**FIGURE 3:**
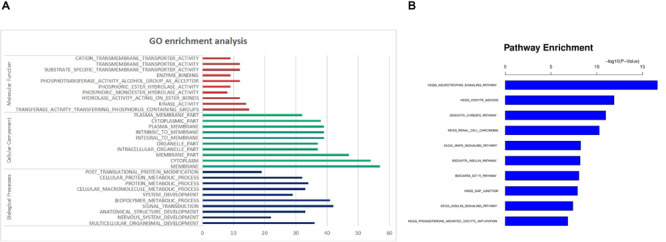
Functional Prediction of DE miRNAs in SNI. **(A)** GO enrichment analysis. **(B)** KEGG pathway analysis.

In the GO enrichment analysis, a total of 90 GO terms were significantly enriched. Thirty GO terms were mainly related to biological processes, such as multicellular organismal development, nervous system development and anatomical structure development. Thirty GO terms were related to cellular components, such as membrane, cytoplasm and intracellular organelle. And thirty GO terms were mainly related to molecular function, such as transferase activity, kinase activity, hydrolase activity and phosphoric ester hydrolase activity. The significantly enriched GO terms are shown in Figure 3A and Supplementary Table S1.

In the KEGG pathways analysis, approximately 10 significant pathways were detected and are shown in Figure 3B and Table 3. Notably, most genes were associated with neurotrophin signaling, oocyte meiosis, biocarta chrebp2, renal cell carcinoma and the mitogen activated kinase-like protein (MAPK) signaling pathway followed by the biocarta insulin, biocarta insulin like growth factor 1 receptor (IGF1R), gap junction, insulin signaling and progesterone mediated oocyte maturation pathways.

**TABLE 3:** KEGG Pathway analysis of DE miRNAs.

Gene set name	Gene number	*p*-Value	Gene name
KEGG_NEUROTROPHIN_SIGNALING_PATHWAY	17	2.36E-17	CRKL,IRAK2,BDNF,MAP2K1,NTF3,YWHAZ,ARHGDIA,YWHAG,IRS1, JUN,PIK3R2,YWHAH,YWHAE,RAF1,YWHAQ,MAPK3,YWHAB
KEGG_OOCYTE_MEIOSIS	13	1.29E-12	PPP2CA,YWHAG,PPP3R1,MAP2K1,BTRC,YWHAH,YWHAE,MAPK3, YWHAB,YWHAZ,YWHAQ,ADCY3,PPP2R1A
BIOCARTA_CHREBP2_PATHWAY	9	1.06E-11	PPP2R1A,PRKAR2A,YWHAZ,YWHAQ,YWHAB,YWHAE,YWHAH, YWHAG,PPP2CA
KEGG_RENAL_CELL_CARCINOMA	10	5.31E-11	VEGFA,EGLN3,RAF1,ETS1,MAPK3,PIK3R2,JUN,MAP2K1,CRKL,TCEB1
KEGG_MAPK_SIGNALING_PATHWAY	14	5.84E-09	PPM1B,PTPRR,RAF1,MAPK3,CACNB2,DUSP1,JUN,MAP2K1, CACNA1C,NTF3,CRKL,BDNF,MAP3K12,PPP3R1
BIOCARTA_INSULIN_PATHWAY	6	6.6E-09	RAF1,MAPK3,IRS1,JUN,MAP2K1,INSR
BIOCARTA_IGF1R_PATHWAY	6	8.88E-09	IRS1,MAP2K1,PRKAR2A,YWHAH,RAF1,MAPK3
KEGG_GAP_JUNCTION	9	1.26E-08	MAPK3,RAF1,LPAR1,TUBB3,GNAI2,ADCY3,TUBA1A,GNAI3,MAP2K1
KEGG_INSULIN_SIGNALING_PATHWAY	10	0.000000041	PRKAR2A,INSR,RAF1,MAPK3,PIK3R2,IRS1,MAP2K1,RPS6KB1,CRKL,CBLB
KEGG_PROGESTERONE_MEDIATED_OOCYTE_ MATURATION	8	0.000000145	GNAI2,ADCY3,RAF1,MAPK3,PIK3R2,MAP2K1,CDC25A,GNAI3

### Network Analysis of DE mRNAs and miRNAs

**FIGURE 4:**
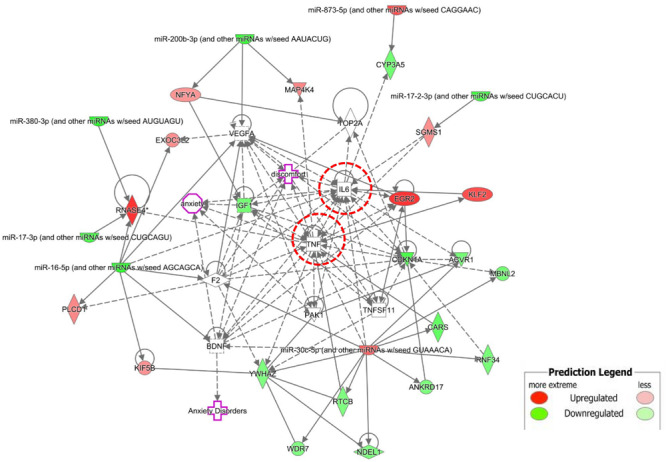
The regulation network was built by Ingenuity Pathway Analysis (IPA). IL-6 and TNF were most associated mRNAs. Mir-30c-5p and mir-16-5p were most connected miRNAs. There were some other core genes in the network, including BDNF, CDKN1a, Egr2 and VEGFA.

To examine the molecular mechanism involved in NP-related anxiety pathogenesis, we performed a regulatory network analysis of the mRNAs and miRNAs involved in SNI pathogenesis. miRNAs were at the center, mRNAs were the target, and the regulation network was built by ingenuity pathway analysis (IPA) (Figure 4). Only experimentally observed microRNA-target interactions and those predicted with high confidence were used. These results illustrated the regulatory relationship between mRNA and miRNA in the mechanism of NP-related anxiety. The network showed that interleukin 6 (IL-6) and tumor necrosis factor (TNF) were most associated with the DE mRNAs and miRNAs, which suggested that inflammatory factors might play an important role in chronic pain with anxiety. Mir-30c-5p and mir-16-5p were the most connected miRNAs in the network. There were some other core genes in the network, including brain derived neurotrophic factor (BDNF), cyclin dependent kinase inhibitor 1A (CDKN1a), early growth response 2 (Egr2) and vascular endothelial growth factor A (VEGFA). Indeed, the regulatory role of mRNAs and miRNAs in anxiety pathogenesis is very complicated, so an in-depth study is still needed in the future.

### qRT-PCR and Western Blot Validation of mRNA Expression

**FIGURE 5:**
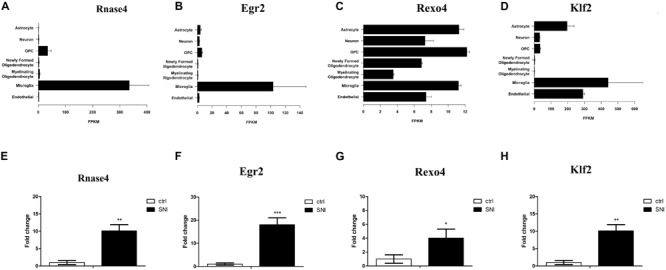
Real-Time Quantitative Polymerase Chain Reaction (qPCR) Validation of key DE mRNAs Expression. **(A–D)** Based on an RNA-Seq transcriptome and splicing database of the cerebral cortex, four genes (Rnase4, Egr2, Rexo4, and Klf2) with significantly increased expression were found abundantly expressed in microglia. The expression level estimation was reported as fragments per kilobase of transcript sequence per million mapped fragments (FPKM) value together with confidence intervals for each sample. **(E–H)** These four mRNAs were analyzed by qPCR, and results were consistent with the results obtained from microarray analysis. *n* = 3 per group; *P < 0.05, **P < 0.01, ***P < 0.001, two-tailed *t*-test. The data are presented as mean ± s.e.m.

Based on an RNA-Seq transcriptome and splicing database of glia, neurons, and vascular cells of the cerebral cortex (Zhang et al., 2014), we searched for the expression of the DE mRNAs in different types of cells. Interestingly, we found that four genes with significantly increased expression were abundantly expressed in microglia, including ribonuclease A family member 4 (Rnase4), Egr2, REX4 homolog, 3′-5′ exonuclease (Rexo4) and Kruppel like factor 2 (Klf2) (Figures 5A–D). These four mRNAs were analyzed by qPCR to validate the reliability of the microarray results and to provide the research a basis for further study. The expression of all of the validated mRNAs was consistent with the microarray results (Figures 5E–H). Western blot was then performed to verify the two most significant genes Rnase4 and Egr2 of the four differential genes. In the PL of the SNI model 14 days after surgery, the protein levels of Rnase4 and Egr2 were significantly reduced when compared with control group (Supplementary Figure S2).

### The Four DE mRNAs Changed Significantly in Microglial M1 and M2 Polarization

**FIGURE 6:**
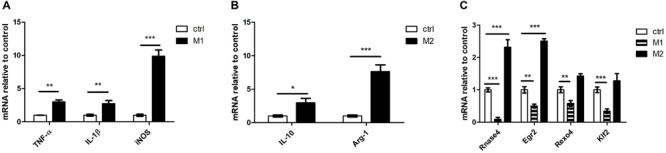
The four DE mRNAs expression change in microglial M1 and M2 polarization. **(A)** qPCR for M1 polarization (TNF-α, IL-1β and iNOS) markers in LPS-stimulated N9 cells. **(B)** qPCR for M2 polarization (IL-10 and Arg-1) markers in IL-4-stimulated N9 cells. **(C)** Rnase4, Egr2, Rexo4, and Klf2 were analyzed by qPCR in microglial M1 and M2 polarization. *n* = 3 per group; *P < 0.05, **P < 0.01, ***P < 0.001, two-tailed *t*-test. The data are presented as mean ± s.e.m.

Microglia can be polarized toward an M1 or M2 phenotype depending on the local microenvironment (Colton, 2009). We chose LPS stimulation to induce microglial M1 polarization and chose IL-4 stimulation to induce microglial M2 polarization in N9 cells. We used TNF-α, IL-1β and inducible nitric oxide synthase (iNOS) as M1 markers and used IL-10 and arginase 1 (Arg-1) as M2 markers. There was a significant increase in TNF-α, IL-1β and iNOS expression following LPS stimulation (Figure 6A). The expression of IL-10 and Arg-1 was significantly increased following IL-4 stimulation (Figure 6B). Furthermore, we detected four DE mRNAs (Rnase4, Egr2, Rexo4 and Klf2) between M1- and M2-polarized macrophages. Our results showed that the expression of all four genes (Rnase4, Egr2, Rexo4 and Klf2) was significantly decreased in M1 polarization conditions and that Rnase4 and Egr2 expression was significantly increased in M2 polarization conditions (Figure 6C).

## Discussion

To our knowledge, this is the first study to screen DE mRNAs and miRNAs in the PL of SNI rats. The DE mRNA-miRNA network showed that IL-6 and TNF were in the central position, and mir-30c-5p and mir-16-5p were the most connected miRNAs in the network. Four genes (Rnase4, Egr2, Rexo4 and Klf2) with significantly increased expression were abundantly expressed in microglia. Furthermore, Rnase 4 and Egr2 expression was decreased in M1-polarized macrophages and increased in M2-polarized macrophages, suggesting their potential role in inhibiting inflammation.

In the healthy brain and spinal cord, microglia represent approximately 10% of CNS cells, which were once virtually ignored. By applying new technologies, more information about the roles of microglia has been obtained, which is growing at an ever-accelerating rate (Salter and Stevens, 2017). Research from Sorge et al. (2015) showed that pain depends on microglial signaling only in males. In this research, we used male SD rats and the details of this signaling mechanism remain to be determined.

Increasing studies have focused on the microglia in the peripheral NP (Beggs et al., 2012; Sorge et al., 2012). It was suggested that in spinal microglia, the activation of P2X4 receptors (P2X4Rs) led to a release of BDNF that subsequently acted on neurons to suppress inhibition, which eventually caused pain hypersensitivity (Masuda et al., 2016). Moreover, in 2016, the study by Li et al. (2016) provided the first evidence that spinal and brain microglia/macrophages had various functions in chronic neuropathic pain. Microglia regulated the function of neurons and synaptic plasticity by releasing inflammatory cytokines and molecules, such as interleukins or TNF (Salter and Stevens, 2017). In the current study, IL-6 and TNF were in the central positions of the DE mRNA-miRNA network, which indicated that microglia were activated in the PL of NP rats.

Microglia have multiple activation phenotypes with different molecular phenotypes and functions that depend on their local microenvironment (Colton, 2009). For example, the M1 phenotype, with high levels of proinflammatory cytokines and oxidative metabolites, can be induced by lipopolysaccharide (LPS) or interferon gamma (IFNγ) (Lynch, 2009). The M1 phenotype is regard as a neurotoxic phenotype due to it may cause tissue destruction or injury. Conversely, IL-4 or IL-10 (anti-inflammatory cytokines) promotes the M2 phenotype, which plays a key role in repairing processes and suppressing destructive immune responses (Ponomarev et al., 2007; Colton, 2009).

Imbalanced microglial polarization to the M1 phenotype may lead to the development of pain, while shift of polarization of microglia to the M2 phenotype may contribute to the relief of pain (Pannell et al., 2016; Huo et al., 2018). Based on an RNA-Seq transcriptome and splicing database of glia, neurons, and vascular cells of the cerebral cortex (Zhang et al., 2014), we found that 4 DE mRNAs (Rnase4, Egr2, Rexo4 and Klf2) with significantly increased expression were abundantly expressed in microglia. Furthermore, the expression of all four genes was significantly decreased in M1-polarized cells, and Rnase4 and Egr2 expression was significantly increased in M2-polarized cells, suggesting that they potentially play an anti-inflammatory role.

In previous research, Egr2 expression was shown to be associated with M2 macrophage polarization (Veremeyko et al., 2018). Moreover, Egr2 labeled more M2 macrophages than the common M2 macrophage marker Arg-1 (Jablonski et al., 2015). Klf2, a nuclear transcription factor known to inhibit inflammation in endothelial cells and monocytes, was also involved in M2 macrophages (van Tits et al., 2011). The downregulation of Klf2 expression in M2 macrophages could lead to a significant increase in macrophage cationic peptide 1(MCP-1) secretion induced by LPS (van Tits et al., 2011). Our results showed that Egr2 and Klf2 expression was significantly decreased in M1-polarized macrophages and Egr2 expression was significantly increased in M2-polarized macrophages, which added new evidence for their role in M2 polarization.

Previous study showed that the expression of microglia M1 markers increases in PFC of rats on day 14 after SNI (Xu et al., 2017). Consistent with their results, Western blot results in current study showed the protein levels of Rnase4 and Egr2 were significantly reduced. However, Rnase4 and Egr2 mRNA were increased in PL of rats on day 14 after SNI. The polarization states of M1 and M2 of PFC may change at different times after SNI surgery (Li et al., 2016). In the PFC of SNI rats, the repairing effect of Rnase4 and Egr2 may start with an increase of mRNA expression before an increase of protein level. On day 14 after SNI, the increase in protein levels had not yet occurred. Chronic pain may also affect mRNA translation and protein modification processes, resulting in inconsistent changes in protein levels and mRNA. Subsequent studies need to use the SNI model for a longer time to verify whether the protein levels of Rnase4 and Egr2 increase during the recovery period. In the present study, we did not verify the role of differential genes in chronic pain or anxiety one by one, which we will continue to complete. In addition, the exact reasons of the differences in protein and gene expression (Rnase4 and Egr2) need to be verified by future studies.

Xu et al. (2017) showed that SNI increased CD68 and iNOS (M1 markers) expression significantly but failed to affect CD206, IL-4 or Arg-1 (M2 markers) expression in the prefrontal cortex. In fact, the negative M2 polarization result might be related to the choice of markers. Our results and previous literature suggested that M2 polarization might have other important markers. In addition, M2 polarization markers might be various in different regions (Tan et al., 2020).

In the DE mRNA-miRNA network, mir-30c-5p and mir-16-5p were the most connected miRNAs. Many previous studies have suggested that they were associated with M2 polarization (anti-inflammatory). Yang et al. (2020) showed that the M2 polarization of tumor-associated macrophages could be promoted by long noncoding RNA (lncRNA) RP11-361F15.2 through miR-30c-5p. A miR-30c-5p agomir might contribute to the transformation of M1 macrophages to M2 macrophages, resulting in changes in inflammatory cytokine levels (Zhang et al., 2019). A recent study showed that miR-16-5p mimics significantly decreased the mRNA expression of IL-1β, IL-6, and TNF-α under LPS stimulation conditions, which indicated that miR-16-5p might be a critical factor involved in the anti-inflammatory effects (Yamada et al., 2020). In another study, lncRNA small nucleolar RNA host gene 1 (SNHG1) reduced inflammation by activating the miR-16-5p-mediated p38 MAPK and the nuclear factor-κB (NF-κB) signaling pathways (Lei et al., 2019). However, the anti-inflammatory function of mir-30c-5p and mir-16-5p in the PL of chronic pain model still needs more research.

In conclusion, we screened dozens of DE mRNAs and miRNAs in the PL of SNI rats. The core of the DE mRNA and miRNA network pointed to molecules associated with inflammation (IL-6, TNF, miR-30c-5p and miR-16-5p). Four mRNAs (Rnase4, Egr2, Rexo4 and Klf2) with significantly increased expression were abundantly expressed in microglia and may be potential markers of M2 polarization. The mechanisms of these inflammation-related molecules in chronic pain need further study.

## Data Availability Statement

The microarray data was deposited in the GEO repository (GSE145226, mRNA and GSE145199, miRNA).

## Ethics Statement

All experimental procedures were approved by the Institutional Animal Care and Use Committee (IACUC) at the Fourth Military Medical University and conformed to the Guide for the Care and Use of Laboratory Animals published by the National Institutes of Health (NIH). Every effort has been made to minimize animal suffering and reduce the number of animals used.

## Author Contributions

SW and JH conceived and designed the experiments. GC, YuZ, YaZ, JC, CG, and FW performed the experiments. GC, YuZ, YaZ, and CG interpreted the data and prepared the figures. SW, JH, and GC wrote and revised the manuscript.

## Conflict of Interest

The authors declare that the research was conducted in the absence of any commercial or financial relationships that could be construed as a potential conflict of interest.

## Footnotes

1 www.ingenuity.com

## Supplementary Material

FIGURE S1 The neuronal loss caused by injection of quinolinic acid into the PL. **(A)** Injection area of quinolinic acid (QA) in the PL (DAPI counterstaining). **(B)** High magnification images showing the immunostaining of glial fibrillary acidic protein (GFAP, green) around the QA injection site in the PL. **(C)** The immunostaining of NeuN (green) around the QA injection site in the PL (adjacent section of A). Bregma 3.80 mm. Scale bar: A, 500 μm; B and C 50 μm.

FIGURE S2 Validation of Rnase4 and Egr2 protein Expression. **(A)** Western blot analysis of Rnase4 and Egr2 in the PL of SNI rats 14 days after surgery. **(B,C)** Rnase4 and Egr2 protein levels were reduced in SNI group. *n* = 3 per group; ^∗^*P* < 0.05, ^∗∗^*P* < 0.01, two-tailed *t*-test. The data are presented as mean ± s.e.m.
