# Supplementary figures and images for: Network Analysis of miRNA and mRNA Changes in the Prelimbic Cortex of Rats With Chronic Neuropathic Pain: Pointing to Inflammation
Source: Front Genet. 2020 Jun 23;11:612. doi: 10.3389/fgene.2020.00612 (PMC7324672; doi:10.3389/fgene.2020.00612)

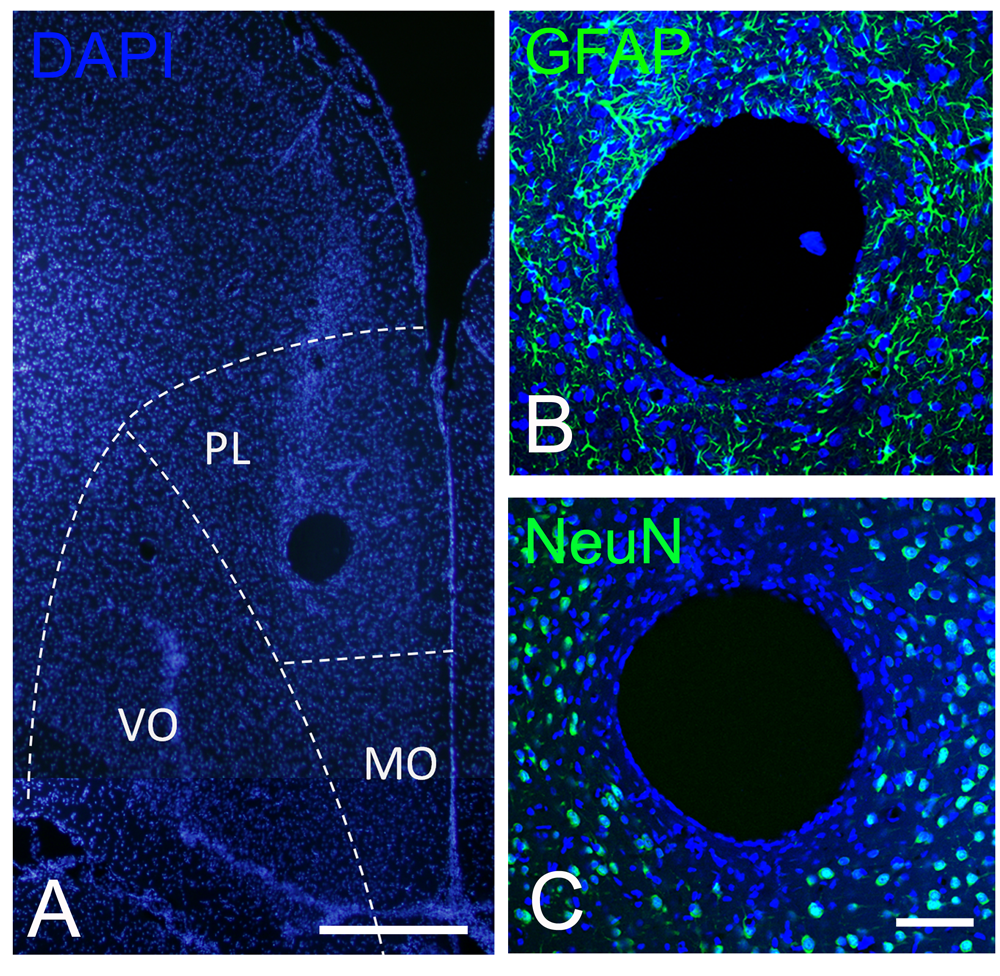

Supplement: FIGURE S1 — The neuronal loss caused by injection of quinolinic acid into the PL. (A) Injection area of quinolinic acid (QA) in the PL (DAPI counterstaining). (B) High magnification images showing the immunostaining of glial fibrillary acidic protein (GFAP, green) around the QA injection site in the PL. (C) The immunostaining of NeuN (green) around the QA injection site in the PL (adjacent section of A). Bregma 3.80 mm. Scale bar: A, 500 μm; B and C 50 μm. [file Image_1.TIF]

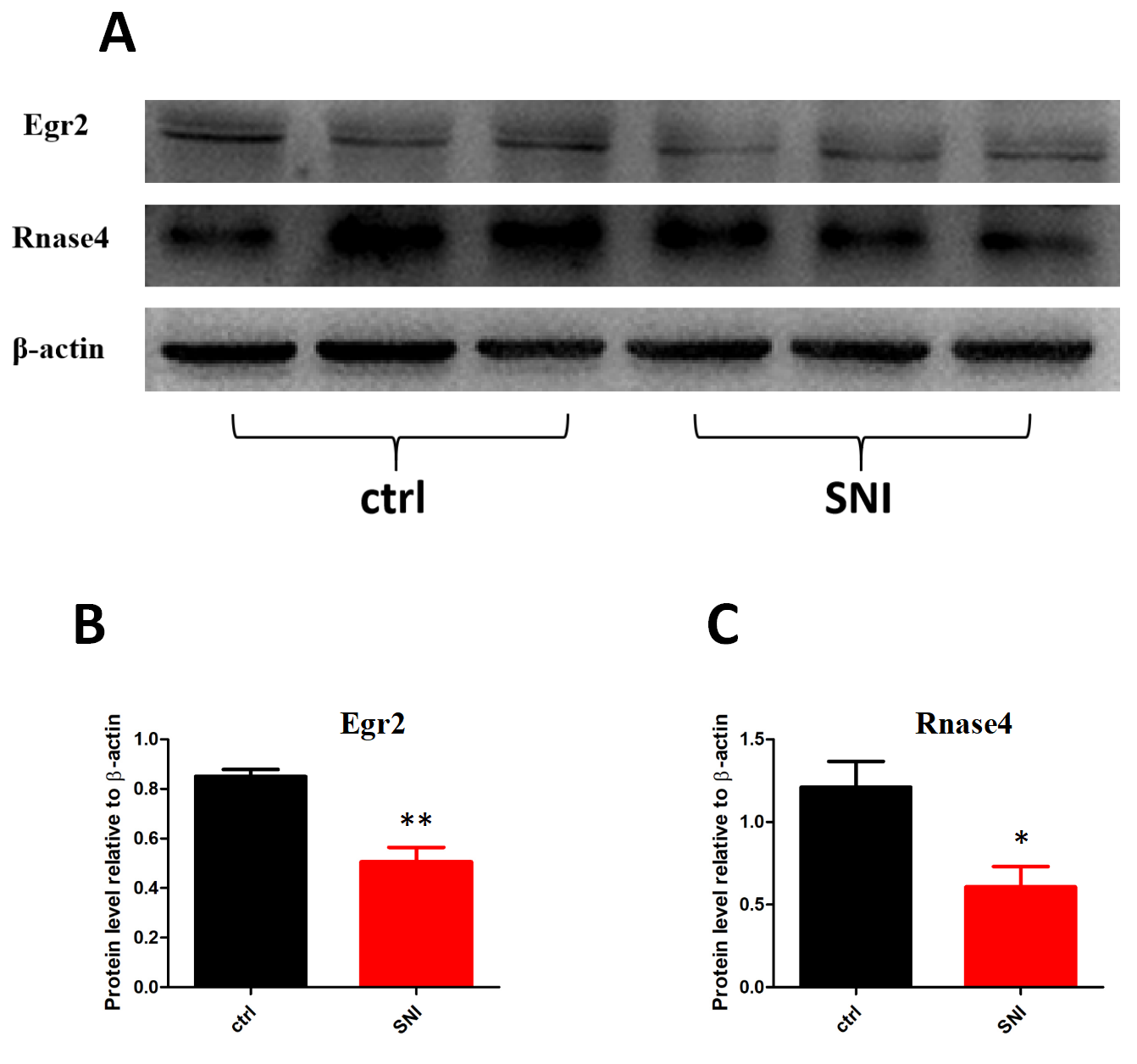

Supplement: FIGURE S2 — Validation of Rnase4 and Egr2 protein Expression. (A) Western blot analysis of Rnase4 and Egr2 in the PL of SNI rats 14 days after surgery. (B,C) Rnase4 and Egr2 protein levels were reduced in SNI group. n = 3 per group; ∗P < 0.05, ∗∗P < 0.01, two-tailed t-test. The data are presented as mean ± s.e.m. [file Image_2.TIF]
